# Smart article: a scientific crosstalk

**DOI:** 10.3389/fphys.2013.00161

**Published:** 2013-06-28

**Authors:** Vahid Haghpanah, Marjan Saeedi

**Affiliations:** Endocrinology and Metabolism Research Center, Endocrinology and Metabolism Clinical Sciences Institute, Endocrinology and Metabolism Research Institute, Tehran University of Medical SciencesTehran, Iran

Modern science is fast-moving, and no laboratory can exist for long with a program based on old facilities. Innovation and renewal are required to keep a laboratory on the frontiers of science (Burton Richter).

An era of the huge expansion of the academic population which is accompanied by the extremely high scientific productivity inevitably causes confusion for scientists who can hardly read all the publications about their topic of interest (Godlee et al., 2000). In the 1980s, a decade which witnessed the explosion of technology, everybody was amazed at the evolution in the research, but nobody could envisage that this evolution would bring its own difficulties. This overwhelming flood of published material may be suggestive of the need for scientists to change their style of producing scholarly articles. The recent increasing trend of data production may even result in discouraging scholars from reading the works of one another thoroughly. This issue can even be exacerbated by the ever-increasing amount of knowledge production and its getting more accessible via the internet (Godlee et al., 2000; Abbott, 2008).

In coming years, scientific publications will be totally technology driven and print version of journals will become obsolete (Bailey and Pang, 2004; Liben-Nowell and Kleinberg, 2008). For the very genuine reason, the ecology of information should be changed in the future and our suggestion to solve this crisis of abundance of scholarly publishing is “Smart Article.”

Smart article is actually an interactive searchable screen, a big transformation from print to beyond the screen. The content is not merely a digital version of a printed article; it is a totally new way of literary structure and interface transformation.

In fact smart articles are fully organized abstracts with smart backbone comprised of different virtual layers. Although each article is displayed in a single page, details are also available in an interactive question-answer based between man-machine. It means that smart searching of structures and substructures will be facilitated in an enhanced article which leads to mutual understanding between article and reader.

The structure of a smart article will be coherent and each segment or paragraph will be logically followed by another one. Unlike the conventional articles, more detailed information about each part including hypothesis, methods, results and the discussion will be available on the platform and the researcher will be able to find all the data he wants to know about, for example the materials and laboratory instruments with access to video, audio, and expandable images.

Therefore, by reading just one embedded searchable article, the researcher could obtain much more information compared with reading just one conventional article at the same time.

Smart article utilizes digital technology to provide a better research experience. The main objective of this technology is to preserve the message of an article and offer a new way of comprehending the concept in an attractive and unique style and provide researchers with the opportunity to find all the required information on his chosen topic with a simple real-time dialog.
